# Level of Bisphenol A in Follicular Fluid and Serum and Oocyte Morphology in Patients Undergoing IVF Treatment 

**Published:** 2019-09

**Authors:** Seyedeh Mahsa Poormoosavi, Mohammad Amin Behmanesh, Sima Janati, Hosein Najafzadehvarzi

**Affiliations:** 1 Research and Clinical Center for Infertility, Department of Histology, School of Medicine, Dezful University of Medical Sciences, Dezful, Iran; 2 Department of Histology, School of Medicine, Dezful University of Medical Sciences, Dezful, Iran; 3 Department of Obstetrics and Gynecology, School of Medicine, Research and Clinical Center for Infertility, Dezful University of Medical Sciences, Dezful, Iran; 4 Department of pharmacology, Faculty of Medicine, Babol University of Medical Sciences, Babol, Iran

**Keywords:** Bisphenol A, Follicular Fluid, IVF, Oocyte Morphology

## Abstract

**Objective:** To assess the correlation between the levels of BPA in the serum and follicular fluid (FF) using oocyte morphology.

**Materials and methods:** In this cross-sectional research, oocyte, FF, and serum samples were obtained from a sample population consisting of 90 women undergone in vitro fertilization in Ganjavian Hospital in Dezful, Iran during October 2017-March 2018. The ELISA kit was utilized for the measurement of the BPA levels. In addition, oocyte morphology simultaneous with inverted optical microscopy.

**Results:** Follicular fluid BPA levels had no significant effect on MII oocytes (p ≥ 0.05). However, the mean levels of degenerated oocytes and germinal vesicle (GV) were significantly higher in the women with high BPA levels in the FF (p ≤ 0.05). Moreover, the mean counts of MII oocytes and oocytes were significantly higher in the women with serum BPA levels of ≤ 50 ng/ml (p ≤ 0.05), while the mean count of GV oocytes was significantly higher in the women with serum BPA levels of ≥ 150 ng/ml (p ≤ 0.05).

**Conclusion:** According to the results, higher FF BPA levels were associated with the higher counts of GVs and oocytes, while oocytes with higher maturity can be achieved in lower levels of BPA in the serum of patients.

## Introduction

Despite the advancement in the methods of preventing infertility, such as the assisted reproductive technology (ART), there is still a large number of infertile men and women who seek treatment and undergo significant costs (1). According to reports, considerable percentage of infertile couples who are treated with high costs and use of ART procedures do not experience a successful pregnancy (2). 

Quality and oocyte morphology evaluations are the essential components of ART. Oocyte morphology has attracted the attention of many researchers to further describe this technique and redefine its effectiveness due to the need for optimal embryo transfer with maximum competency (3). The components of plasma transfer result in the production of the follicular fluid (FF) through thecal secretions, as well as the blood-follicle barrier from the granulosa cells (4). FF is a microenvironment that plays a pivotal role in the production and quality of oocytes, as well as the somatic cells that encircle FF via direct contact (5). The development potential and viability of oocytes are influenced by specific compositions of FF (5). 

Pollutants are composed of various hazardous chemical and their metabolites, which affect the health of humans on a daily basis (6). Bisphenol A (BPA), with the chemical formula of 4,4-dihydroxy-2,2-diphenylpropan, is utilized as a raw material in the production of polycarbonate plastics and epoxy resins, which are key elements in various sectors and industries, such as food and beverage packaging, dentistry, and water pipes (7). 

BPA was first developed as a synthetic estrogen as it is able to bind to estrogen receptors. However, the genes activated by BPA differ from those stimulated by estradiol (8). Animal studies have indicated that BPA exposure adversely affects the reproductive system through histological changes in the tract (9). According to the literature, BPA measurement and leaching are possible in human serum, amniotic fluid, urine, umbilical cord blood, and placental tissues (10). 

Despite the large number of the animal experiments in this regard, only few human studies have been focused on the measurement of the BPA levels in various body fluids, such as the FF (11). Furthermore, data is scarce regarding the exact effects of chemicals such as BPA, which impair the endocrine system, on oocyte morphology in human subjects.

The present study aimed to assess FF and serum BPA levels and comparisons of the means of these levels and oocyte maturation.

## Materials and methods

Using a cross-sectional design, 90 women aged 20-45 years were selected from the patient population in Omolbanin Infertility Center of Ganjavian Hospital in Dezful, Iran during October 2017-March 2018. The selected subjects received ART for infertility. Data were collected via enquiries, including the age and causes/duration of infertility. The records of the patients were reviewed, and the level of estradiol was extracted. In addition, height, body weight, and body mass index (women) were measured.

The research protocol was approved by the Ethics Committee of Dezful University (IR.DUMS.REC.1395.7). Written informed consent was obtained from all the participants prior to the study.

Women with polycystic ovarian syndrome, endocrine, metabolic or infectious disorder, endometriosis, FSH > 12, ovarian factor and hypothalamic amenorrhea.

In all patient, for pituitary down-regulation and endogenous gonadotropin depletion, according to the long agonist protocol, starting daily injection of 0.5 mg subcutaneous Busereline (Superfact; Aventis, Frankfurt, Germany) on day 21 of the menstrual cycle preceding gonadotropin treatment. The dose was reduced to 0.25 mg daily on the second day of menstruation until the day of hCG Administration. As menses started, gonadotropin stimulation was recommended to be done by means of Gonal-F (Gonal-F, Serono, Aubnne, Switzerland) from the second day of the menstrual period. According to the patients’ age and body weight, the initial dose of gonadotropin was determined to be 150-300 IU/day (12). 

Serial ultrasound monitoring was started on day 7 of the stimulation, and evaluation of serum estradiol (E2) levels were used to assess ovarian response, and then gonadotropin dose adjustments were done as required. Human chorionic gonadotropin (Pregnyl, Organon, Oss, and the Netherlands) 10,000 IU intramuscular (IM) was administered when at least three follicles reached a mean diameter of ≥18 mm. 

Oocyte retrieval was performed 34-36 h after hCG administration under ultrasound guidelines in lithotomy position. Vaginal ultrasound was carried out for follicle extraction at two stages of examination and suctioning. Puncturing was performed for the removal of oocytes from the ovaries, and a minimum follicular diameter of 18 millimeters and FF were defined for aspiration by catheter. The obtained samples were transferred to test tubes. G-MOPS medium (Vitrolife-Sweden) was used to wash the oocytes, and the oocytes were incubated for two hours at the temperature of 37°C with 6% CO_2_. When the oocytes were removed, the FF samples were centrifuged at 3,000 rpm for 10 minutes to pellet the follicular cells. At the final stage, only the supernatant of the blood-free samples was aliquoted into two milliliters of cryovials and stored at the temperature of -70°C until the assays (13).

The oocytes were classified into three categories using an inverted microscope (Olympus). The categories were as follows: metaphase II (MII) (presence of first polar body), metaphase I (MI) (absence of first polar body and germinal vesicle breakdown) germinal vesicle (GV), and degenerated oocytes (14). 

One milliliter of FF was sampled from each patient. The ELISA kit was used for the measurement of the BPA levels (Catalog#MBS2602664, MyBiosource Company). It is notable that we utilized the double-sandwich ELISA technique. General BPA monoclonal antibody was considered as the pre-coated antibody, and the detecting antibody was biotin-labeled polyclonal antibody. The biotin-labeled antibody was added to the ELISA plate wells and washed out with phosphate-buffered saline (PBS). 

At the next stage, the ELISA wells were filled with avidin peroxidase conjugates in an orderly fashion. We also used the 3,3`,5,5`-Tetramethylbenzidine (TMB) substrate for staining after the reactant, which was thoroughly washed using PBS. Following that, TMB was converted into blue using catalytic peroxidase and then converted into yellow as a result of acidity. Positive correlations were denoted between the color depth and testing factors in the samples (15).

Data analysis was performed in SPSS version 22 using Fisher’s least significant difference (LSD) in order to determine the significant differences between the sample groups and one-way analysis of variance (ANOVA) to compare the variances of the groups. In all the statistical analyses, P-value of ≤ 0.05 was considered significant.

## Results

***Patients and Oocytes:*** In total, 90 women were enrolled in the current research, and ovarian stimulation oocytes were collected from the participants. Mean oocyte count was 6.5 ± 3.21 (range: 1-30). Mean count of MII oocytes, MI oocytes, and GV and degenerated oocytes was estimated at 3.01 ± 2.1, 3.4 ± 1.3, and 2 ± 1.1, respectively.

***Comparison of Study Groups in Terms of Oocyte Morphology:*** According to the results of the present study, the mean count of MII oocytes was significantly higher in the women aged less than 30 years (p ≤ 0.05), as well as those with lower body mass index (p ≤ 0.05) and higher serum estradiol levels (p ≤ 0.05). However, no significant differences were observed in the mean count of MII oocytes between the study groups in terms of the cause and duration of infertility (p > 0.05) (Table 1).

***BPA Levels in FF and the Association with Oocyte Morphology:*** No significant associations were observed between the BPA level in FF with the counts of oocytes, MII oocytes, and MI oocytes (p > 0.05). The highest mean count of MII oocytes (3.46 ± 5.23) was denoted with the BPA levels of ≤ 50 ng/ml in FF, while the lowest mean count of MII oocytes (1.69 ± 5.41) was observed in the BPA levels of 100-150 ng/ml in FF. 

According to the obtained results, the mean counts of GV and degenerated oocytes were significantly higher in the women with higher BPA levels in FF (p ≤ 0.05) (Table 2). Considering that the minimum level of BPA in FF was estimated at 19.41 ng/ml in the current research, categorization of the samples was initiated at the level of 19.41. Correspondingly, the maximum BPA level was calculated to be 148.62 mg/dl, which was decided to be the maximum limit of the final study group. 

**Table 1:** Comparing average number of MII oocytes and other oocytes at different groups of studied variables

		**Oocytes**	**MII**	**MI**	**GV, Degenerated**
Women age	25-30	14.33 ± 3.29^a^	8.26 ± 3.21^a^	4.66 ± 1.57	1.57 ± 2.62^b^
	30-35	9.35 ± 6.07^b^	2.6 ± 3.48^b^	4.21 ± 2.86	1.7 ± 2.88^b^
	35-40	9.33 ± 2.93^b^	2.5 ± 3.48^b^	5 ± 6.39	2.66 ± 2.08^b^
	40-45	8.35 ± 5.31^b^	0.8 ± 1.78^c^	5.01 ± 2.91	3.6 ± 3.96^a^
	≥45	8.21 ± 1.83^b^	0. 1 ± 4.11^c^	5.3 ± 2.34	3.01 ± 3.11^a^
Women BMI	< 25	10.62 ± 2.72^a^	4.62 ± 1.56^a^	3.25 ± 1.25	1.95 ± 0.64^a^
	25-30	8.56 ± 3.98^b^	1.52 ± 2.66^b^	5.31 ± 1.2	2.74 ± 2.71^b^
	> 30	7.75 ± 2.46^b^	0.5 ± 2.51^b^	5.76 ± 2.54	5.41 ± 2.73^b^
Cause of infertility	Male factor	11.62 ± 3.42^a^	4.17 ± 2.32	4.11 ± 4.5	2.44 ± 2.01
	Female factor	7.31 ± 3.2^b^	3.01 ± 2.66	3.4 ± 4.13	1.13 ± 2.99
	Both	7.04 ± 5.3^b^	2.5 ± 2.51	3.1 ± 3.57	1.67 ± 2.57
Estradiol (pg/ml)	< 2000	8.65 ± 6.43^b^	3.46 ± 3.5^b^	3.35 ± 5.09	2.89 ± 2.95
	2000-4000	9.11 ± 6.56^b^	3.21 ± 2.78^b^	4.22 ± 5.99	2.29 ± 3.63
	4000-6000	15.33 ± 4.09^a^	7.78 ± 2.18^a^	5.33 ± 2.52	3.33 ± 2.25
	6000-8000	16.01 ± 5.96^a^	8.02 ± 3.14^a^	5.02 ± 1.73	3.2 ± 2.29
Infertility duration (Year)	≤ 5	8.08 ± 2.9	2.25 ± 2.27	3.75 ± 1.91	1.33 ± 1.95
	> 5	9.47 ± 4.69	2.23 ± 1.06	4.82 ± 3.04	2.46 ± 2.68

a, b and c in each column indicate significant differences at p ≤ 0.05.

**Table 2:** Comparing the average distribution of MII oocytes and other oocytes between different levels of BPA in FF and serum

**BPA level ng/ml**	**Oocytes**	**MII**	**MI**	**GV, Degenerated**
Follicular fluid				
≤ 50	9.13 ± 5.34	3.46 ± 5.23	3.86 ± 4.8	1.8 ± 2.87^b^
50- 100	8.12 ± 6.32	2.37 ± 3.82	4.6 ± 2.32	2.11 ± 3.38^b^
100- 150	10.02 ± 4.69	1.69 ± 5.41	5.75 ± 3.59	6.59 ± 1.79^a^
≥ 150	13.66 ± 5.65	2.46 ± 4.76	4.21 ± 3.99	6.88 ± 2.64^a^
Serum				
≤ 50	11.81 ± 6.42^a^	6.18 ± 5.07^a^	4.62 ± 3.55	2.01 ± 2.83^b^
50- 100	6.01 ± 3.59^b^	1.5 ± 3.02^b^	3.52 ± 2.38	1.75 ± 5.11^b^
100- 150	12.1 ± 3.59^a^	1.8 ± 5.94^b^	5.83 ± 3.18	7.16 ± 2.9^b^
≥ 150	13.23 ± 5.33^a^	2.02 ± 5.65^b^	4.91 ± 5.65	8.01 ± 1.74^a^

a, b Significant differences at p ≤ 0.05.

***Serum BPA Level and the Association with Oocyte Morphology:*** According to the present study, the mean counts of oocytes (11.81 ± 6.42) and MII oocytes (6.18 ± 5.07) were significantly higher in the women with the serum BPA levels of ≤ 50 ng/ml (p ≤ 0.05). Furthermore, the mean count of GV oocytes (8.01 ± 1.74) was found to be significantly higher in the women with the serum BPA levels of ≥ 150 ng/ml (p ≤ 0.05) (Table 2). Considering that the minimum serum BPA level was 4.62 ng/ml in the samples evaluated in the current research, categorization of the samples was initiated at the level of 4.62. It is also notable that the maximum level of BPA was calculated to be 149.92 ng/ml, which was decided to be the maximum limit of the final study group.

## Discussion

BPA has widely been reported to impair the endocrine system, while it also plays a disruptive role in the reproductive system. The debilitative effects of BPA on the reproductive organs of mammals have been well established in the current literature (16). FF contributes to the regeneration of oocytes, as well as the adjustment of various components of FF that are associated with the quality of oocytes (17). As such, the quality and maturation of FF is largely influenced by cell changes. In the current research, we initially examined the correlation between the levels of BPA in the FF, as well as the serum levels of this hazardous chemical. Following that, the associations between these parameters and oocyte morphology and quality were investigated. 

In the present study, the maximum mean count of MII oocytes was denoted with the serum BPA levels of ≤50 ng/dl. However, no significant association was observed between the FF levels of BPA and MII oocyte count. In addition, the highest mean counts of GV and degenerated oocytes were denoted at the maximum serum and FF levels of BPA. Therefore, the mechanisms involved in the mentioned effects of BPA on human oocytes could only be speculated. 

According to Hunt et al. (18), a significant increase in meiotic abnormalities due to coincidental BPA exposure is the primary indicator of the debilitative effects of BPA on the development of the mentioned oocytes. In this regard, our findings are in congruence with the study by Lenie et al. (19), which denoted that BPA caused changes in the murine oocyte development through inducing greater meiotic arrest at the GV or metaphase I stages, which only occurred at the highest concentration of 30 mmol/L.

According to the findings of Zhang et al. (9), the deterioration of oocyte quality due to BPA in mice occurred as a consequence of oxidative stress (20). Furthermore, it was proposed that the melatonin dosage of 30 mg/kg of the body weight of the animals (2.5 mg in humans) could minimize the damages caused to human oocytes following BPA exposure. 

In another experiment in this regard, Deyanira Guadalupe et al. (18) claimed that BPA accelerate meiotic progression, thereby impairing prophase I to the metaphase II transition. This mechanism is associated with the reduction of bidirectional communication in the cumulus cell-oocyte complex. On the same note, the findings of Peretz et al. demonstrated the impact of BPA on steroidogenesis in the granulosa and theca cells, and the process initiated through the down-regulation of StAR, followed by the effects on the granulosa-theca cell communication (e.g., hormone diffusion from the theca cells into the granulosa cells). 

Impaired follicle growth in the antral follicles could be a direct consequence of malfunctioning granulosa cells since the proliferation of these cells has been reported to augment the size of the developing follicles. Furthermore, impaired follicle growth may weaken hormone production as granulosa cells are the only cells in the antral follicle agents to aromatize the androgens in the theca into estrogens (19).

## Conclusion

According to the obtained results, BPA exposure adversely influences the reproductive system in humans. Among our participants, women undergoing *in-vitro* fertilization who had higher BPA concentrations are at the risk of significantly higher BPA levels, as well as a low count of MII oocytes and a high count of GV oocytes. Moreover, BPA exposure exerts debilitative effects of the quality and morphology of oocytes. Therefore, it is recommended that further investigations be conducted regarding the exact mechanisms of the oocyte and embryonic toxicities induced by BPA.
